# Seven new dolphin mitochondrial genomes and a time-calibrated phylogeny of whales

**DOI:** 10.1186/1471-2148-9-20

**Published:** 2009-01-25

**Authors:** Ye Xiong, Matthew C Brandley, Shixia Xu, Kaiya Zhou, Guang Yang

**Affiliations:** 1 Jiangsu Key Laboratory for Biodiversity and Biotechnology, College of Life Sciences, Nanjing Normal University, Nanjing, PR China; 2 Department of Ecology and Evolutionary Biology, Yale University, New Haven, Connecticut, USA

## Abstract

**Background:**

The phylogeny of Cetacea (whales) is not fully resolved with substantial support. The ambiguous and conflicting results of multiple phylogenetic studies may be the result of the use of too little data, phylogenetic methods that do not adequately capture the complex nature of DNA evolution, or both. In addition, there is also evidence that the generic taxonomy of Delphinidae (dolphins) underestimates its diversity. To remedy these problems, we sequenced the complete mitochondrial genomes of seven dolphins and analyzed these data with partitioned Bayesian analyses. Moreover, we incorporate a newly-developed "relaxed" molecular clock to model heterogenous rates of evolution among cetacean lineages.

**Results:**

The "deep" phylogenetic relationships are well supported including the monophyly of Cetacea and Odontoceti. However, there is ambiguity in the phylogenetic affinities of two of the river dolphin clades Platanistidae (Indian River dolphins) and Lipotidae (Yangtze River dolphins). The phylogenetic analyses support a sister relationship between Delphinidae and Monodontidae + Phocoenidae. Additionally, there is statistically significant support for the paraphyly of *Tursiops *(bottlenose dolphins) and *Stenella *(spotted dolphins).

**Conclusion:**

Our phylogenetic analysis of complete mitochondrial genomes using recently developed models of rate autocorrelation resolved the phylogenetic relationships of the major Cetacean lineages with a high degree of confidence. Our results indicate that a rapid radiation of lineages explains the lack of support the placement of Platanistidae and Lipotidae. Moreover, our estimation of molecular divergence dates indicates that these radiations occurred in the Middle to Late Oligocene and Middle Miocene, respectively. Furthermore, by collecting and analyzing seven new mitochondrial genomes, we provide strong evidence that the delphinid genera *Tursiops *and *Stenella *are not monophyletic, and the current taxonomy masks potentially interesting patterns of morphological, physiological, behavioral, and ecological evolution.

## Background

Cetaceans (whales, dolphins, and porpoises) have been the subjects of intense phylogenetic inquiry using both morphological (including fossil) and molecular data [1-19]. This attention is not surprising; Cetaceans represent one of the most fascinating evolutionary transitions within vertebrates and a robust phylogenetic framework is the underpinning of any study into morphological, behavioral, and physiological evolution. The results of these phylogenetic inquiries agree on several key relationships including the monophyly of Cetacea and Mysticeti (baleen whales), and the close relationships of the Amazon (Iniidae) and La Plata (Pontoporidae) River dolphins.

**Figure 1:**
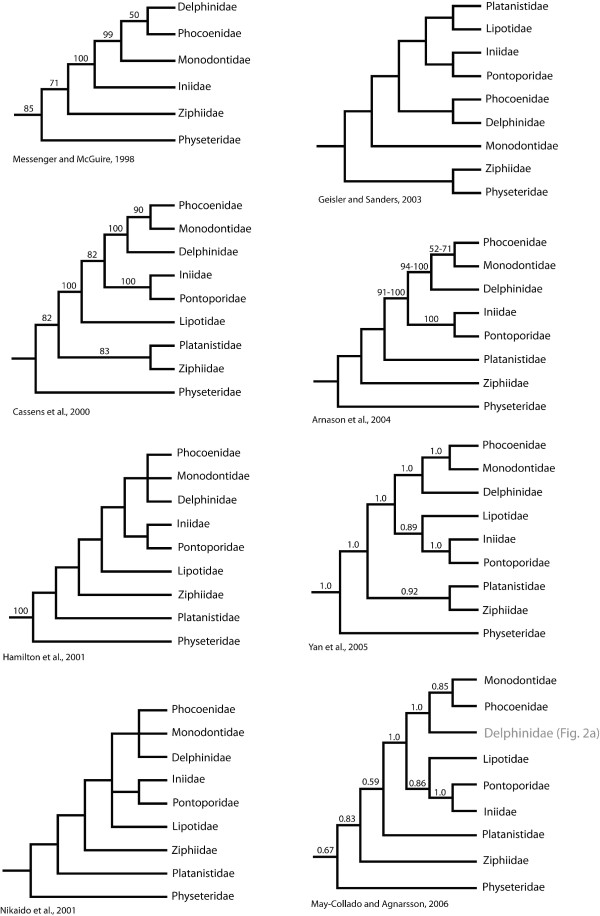
**Recent hypotheses of the interrelationships of the major cetacean lineages**. Clade support values, when available in the original study, are provided. Decimal numbers represent posterior probabilities and numbers between 50 and 100 represent nonparametric bootstrap proportions.

However, many of the phylogenetic relationships inferred by the aforementioned studies strongly conflict. Perhaps the most obvious incongruencies are the interrelationships of the major odontocete (toothed whales) clades (Fig. 1). All published phylogenies disagree on the phylogenetic placement of one or more families, and many of these incongruencies are strongly supported with high bootstrap proportions or posterior probabilities (Fig. 1). Thus, we have little confidence in the "deeper" relationships of odontocetes and this situation limits the power of any comparative analyses that incorporate phylogenetic information.

**Figure 2:**
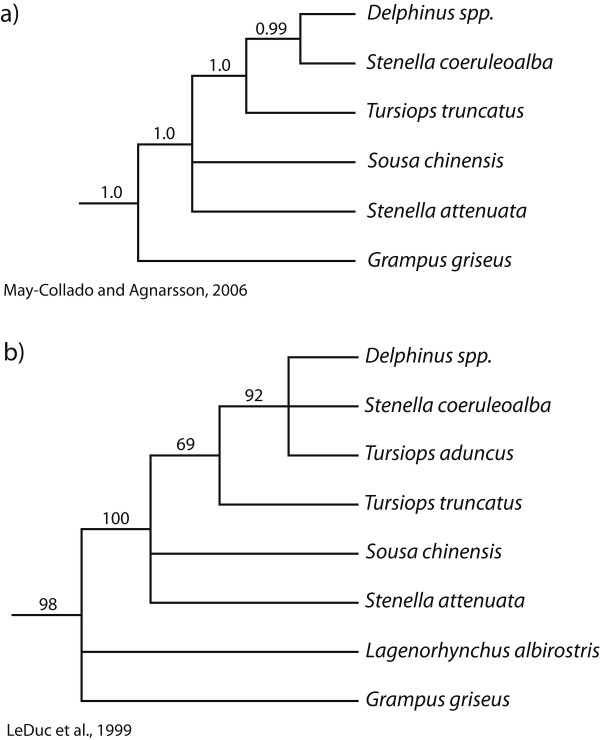
**Recent hypotheses of the interrelationships of selected Delphinidae species**. The original phylogenies were pruned to include only species used in the current study. Decimal numbers represent posterior probabilities and numbers between 50 and 100 represent nonparametric bootstrap proportions.

There is also evidence that the traditional taxonomy of dolphins (Delphinidae) does not adequately capture the diversity of the group, especially within the *Sousa*-*Delphinus*-*Tursiops*-*Stenella *complex (Fig. 2). Bottlenose dolphins (genus *Tursiops*) have been classified into as few as one and as many as eight different species [20-22], but most recent analyses recognize two distinct species, the common bottlenose dolphin (*T. truncatus*), and the Indo-Pacific bottlenose dolphin (*T. aduncus*). The points of contention, however, are the phylogenetic affinities of these two species. Recent molecular evidence lends support to the hypothesis that *T. aduncus *is not only a distinct species, but is more closely related to the striped dolphin (*Stenella coeruleoalba*) than to *T. truncatus *([9], Fig. 2b). This contrasts with osteological similarities suggesting that the two *Tursiops *species are sister taxa [23]. In addition, two molecular phylogenetic studies of Delphinidae inferred strong support for the paraphyly of the genus *Stenella *with respect to *Tursiops *and *Delphinus *([9,17], Fig. 2). Moreover, the phylogenetic relationships of all of these genera with respect to the genus *Sousa *(humpback dolphins) is also unresolved (Fig. 2) (see also [19]).

Thus, it is clear that the overall state of odontocete phylogenetics, and the evolutionary history of delphinids in particular, remains incompletely resolved. There are myriad possible explanations for ambiguous and conflicting results among these phylogenetic studies. One possiblity is that the phylogenetic methods used in these analyses do not adequately capture the complex nature of DNA evolution, thus allowing systematic error to bias the results. Some studies [8,9,12,14,24] have used phylogenetic analyses that are known to be misled by the complexity of DNA evolution (e.g., maximum parsimony; [25-29]) or do not implement an optimality criterion to distinguish between competing hypotheses (e.g., neighbor joining [27,29]). While many studies [5,11,15-17,19,30] have incorporated phylogenetic methods that explicity incorporate molecular evolution parameters including base frequencies, substitution rates, and rate heterogeneity and an optimality criterion (e.g., maximum likelihood and Bayesian), this does not necessarily mean that they have adequately incorporated all of the critical parameters for modeling DNA evolution. For example, until very recently, there have been insufficient methods to incorporate lineage-specific rates of evolution in phylogenetic analyses. Instead, most phylogenetic methods assume that a phylogeny is unrooted, thereby assuming every lineage evolves at an independent rate (e.g., maximum parsimony, most implementations of maximum likelihood and neighbor-joining) or that all lineages evolve at the same rate (e.g., UPGMA and methods enforcing a molecular clock) [31]. However, the recent development of "relaxed" molecular clocks [31] offers a compromise between the two extremes of a global rate and completely independent species-specific rates. The advantage of incorporating this parameter is the same as incorporating any other parameter that makes a phylogenetic model more "realistic"; the better the model, the more accurate the phylogeny. Moreover, use of relaxed molecular clocks and fossil calibration constraints also permits estimation of divergence times of clades. Therefore, the rich fossil history of cetaceans (see [32]), the development of relaxed molecular clocks, and newly available computer programs to implement this advancement in phyogenetic modeling (e.g., BEAST; [33]) may provide powerful new tools to examine the phylogenetic history of cetaceans.

Furthermore, ambiguous relationships among the delphinid *Delphinus*-*Tursiops*-*Stenella *complex inferred by analyses of mitochondrial DNA may be attributable to the relatively small number of nucleotides used by previous phylogenetic analyses (1140 of cytochrome *b *[9,17]). Given the massive number of potentially informative characters (>10,000+ nucleotides) that they contain, complete mitochondrial genomes could potentially resolve the current uncertainty in the phylogeny of these delphinids. While complete genomes exist for all the major families of cetaceans, and have been subsequently used in phylogenetic analyses [5,6,30,34], to date, mitochondrial genomic information is available for only a single delphinid (*Lagenorhynchus albirostris*).

A reanalysis of cetacean phylogeny and *Stenella *and *Tursiops *taxonomy, using recently-developed complex phylogenetic models of DNA evolution and an expanded mitochondrial dataset, is warranted. To this end, we sequenced the complete mitochondrial genomes of seven dolphin species including the common bottlenose dolphin, Indo-Pacific bottlenose dolphin, Long-beaked common dolphin (*Delphinus capensis*), Pantropical spotted dolphin (*Stenella attenuata*), striped dolphin (*St. coeruleoalba*), Risso's dolphin (*Grampus griseus*), and the Indo-Pacific humpback dolphin (*Sousa chinensis*). We analyze these data, and existing cetacean mitochondrial genomes, with Bayesian relaxed clock phylogenetic analyses to address two questions in cetacean phylogenetics. First, how do implementing complex phylogenetic models affect our understanding of the relationships of the major odontocete families? Secondly, does mitochondrial genomic data support the hypotheses that the delphinid genera *Tursiops *and *Stenella *are polyphyletic? Resolution of both questions may have a profound impact of any future comparative biological analyses of odontocetes, and delphinids in particular.

## Results

### Characteristics of the mitochondrial genes

In terms of base compositions, gene structure, and gene order, the characteristics of the mitochondrial genomes of the seven newly sequenced delphinids are very similar to the only other existing delphinid mt genome, that of the white-beaked dolphin, *L. albirostris *[5]. The sizes of the genomes range from 16,384 to 16,393 bp. The base composition of the heavy-strand, excluding the control region, was similar among different mt-genomes, with average values and ranges (in parenthesis) of: A, 31.5% (31.4%–31.7%); C, 28.0% (27.8%–28.3%); G, 11.4% (11.3%–11.6%); and T, 29.0% (28.7%–29.2%). Most genes had standard stop codons, while ND3 and ND4 had incomplete stop codons (TA or T), with the terminal 3' nucleotide being contiguous with the 5' terminal nucleotide of the following tRNA genes. It is assumed that the stop codon is completed with the addition of the poly-A tail [35]. Gene order of all eight available delphinid mt-genomes was consistent and did not deviate from those of other cetaceans or the standard vertebrate gene order.

### Phylogenetic results

**Figure 3:**
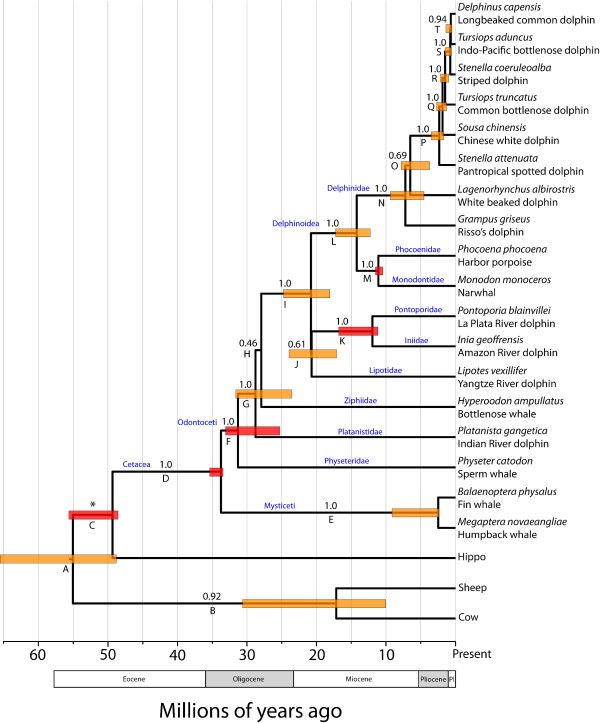
**Chronogram of Cetacea inferred by partitioned Bayesian analyses, enforcing a lognormal relaxed molecular clock, of 12 mitochondrial protein-coding genes**. Numbers above the clades represent Bayesian posterior probabilities. Clade letters refer to Table 1. Red boxes indicate nodes for which a prior calibration constraint distribution was used and orange boxes indicate divergence dates estimated without prior calibration constraints for that node. The bounds of the boxes delimit the 95% highest posterior density (HPD) for the clade age. The asterisk indicates that the monophyly of this group was constrained in the phylogenetic analysis.

The two partitioned Bayesian analyses achieved stationarity by 5 million generations, and posterior distributions of each parameter were calculated for the remaining 15 million post burn-in trees. The phylogeny, and 95% credible intervals of divergence times, are provided in Fig. 3. Overall support for the phylogeny is very high with 13 clades strongly (i.e., statistically) supported. The "deeper" relationships of the phylogeny are all well-supported including the monophyly of Cetacea and Odontoceti (toothed whales). The basal divergence within odontocetes is between the physeteroids (sperm whales) and a strongly supported clade (Clade G; PP = 1.0) of remaining odontocete families. The basal relationships within this clade are weakly supported and essentially form a trichotomy including Platanistidae (Indian River dolphins), Ziphiidae (bottlenose whales), and a strongly supported clade (Clade I; PP = 1.0) including other dolphins and porpoises. The relationship between Lipotidae (Yangtze River dolphin) to the two other river dolphin families Pontoporidae (La Plata River dolphins) and Iniidae (Amazon River dolphins) is only weakly supported (PP = 0.61), but support for the sister relationship of the latter two families is significant (PP = 1.0). The remaining marine dolphins and porpoises form a strongly supported clade (Clade L; PP = 1.0), with a basal divergence between a strongly supported Delphinidae (PP = 1.0) and a clade including Phocoenidae (porpoises) and Monodontidae (narwhals and belugas). The interrelationships of *G. griseus*, *L. albirostris*, and other delphinid genera are not well supported. Within the strongly supported clade of remaining delphinids (Clade P; PP = 1.0), there is statistically significant support for the paraphyly of *Tursiops *and *Stenella*. *Stenella attenuata*, *Sousa chinensis*, and *T. truncatus *form sequentially more exclusive, strongly supported clades (Clades P, Q, and R; PP = 1.0) with the other delphinids. *T. aduncus *(Indo-Pacific bottlenose dolphin) forms a strongly supported clade (PP = 1.0) with *D. capensis *and *St. coeruleoalba*. (but the sister relationship of *D. capensis *and *T. aduncus *is highly but not statistically supported [PP = 0.94]).

The divergence dates inferred by the Bayesian relaxed clock analyses suggest that the major odontocete lineages diversified in Mid- to Late Oligocene (Fig. 3; Table 1). The four river dolphin lineages diverged in distinctly different time periods, with the Indian River dolphins (Platanistidae) diverging Late Oligocene, Yangtze River dolphins (Lipotidae) in the very Late Oligocene or Early Miocene, and La Plata River (Pontoporidae) + Amazon River (Iniidae) dolphins in the Mid-Miocene. The divergence of the three extant delphinoid lineages occurred in the Mid-Miocene. The crown Delphinid lineages radiated in the Late Miocene, with the *Sousa*-*Delphinus*-*Tursiops*-*Stenella *complex diverging recently in the Mid- to Late Pliocene.

**Table 1:** Divergence times of lineages analyzed in this study, estimated from partitioned Bayesian phylogenetic analyses of 12 mitochondrial protein-coding genes using a lognormal relaxed molecular clock.

Clade	Age	Lower 95% HPD	Upper 95% HPD
A	55.04	48.86	65.58

B	17.16	10.10	30.71

C	49.34	48.61	55.71

D	33.74	33.52	35.43

E	2.49	2.46	9.20

F	31.29	25.35	33.20

G	28.77	23.62	31.68

H	27.95	-	-

I	20.78	18.17	24.82

J	20.70	17.18	24.01

K	11.95	11.25	16.88

L	14.21	12.36	17.32

M	11.10	10.51	11.59

N	7.21	4.61	9.46

O	6.50	3.80	7.89

P	2.35	1.77	3.53

Q	1.86	1.36	2.79

R	1.45	1.04	2.26

S	0.77	0.62	1.58

T	0.68	0.59	1.47

Clade letters refer to Fig. 3. HPD = highest posterior density of the age of the clade (HPD not available for node H due to its low posterior probability). Units are in millions of years.

## Discussion

This study is the first to use partitioned Bayesian analyses with models of rate autocorrelation (i.e., the relaxed molecular clock) and complete mitochondrial genomes to elucidate the phylogenetic relationships of Cetacea and age of each clade. Furthermore, we focused our analysis both at the "deep" phylogenetic level, reconstructing the evolutionary relationships among the major extant odontocete lineages, and the "shallow" level examining interrelationships among delphinid species in the *Sousa*-*Delphinus*-*Tursiops*-*Stenella *complex. Our results do much to increase our understanding of cetacean phylogenetic relationships, and our estimation of molecular divergence dates allow us to test previous hypotheses of ancestral divergence events.

### Odontocete relationships and potential explanations for continuing phylogenetic uncertainty

Although numerous studies have demonstrated that Physeteridae (sperm whales) represent the sister lineage to all other odontocetes [5,8,10,11,13,17,30,36], there has been significant disagreement about the phylogenetic affinities of the other odontocete families. Although previous studies have supported various hypotheses (Fig. 1), they can essentially be simplified to disagreements about the placement of the river dolphin families Platanistidae (Indian River dolphins) and Lipotidae (Yangtze River dolphins) and relationships among the delphinoids, Delphinidae (marine dolphins), Monodontidae (narwhals and belugas), and Phocoenidae (porpoises).

Numerous studies have refuted the existence of a single river dolphin clade [5,8,10,13,17,24,30,37-40], and instead have proposed conflicting relationships of the four major river dolphin clades. While most studies strongly support the sister relationship between Iniidae (Amazon River dolphins) and Pontoporidae (La Plata River dolphins), there are numerous strongly supported conflicting hypotheses of evolutionary relationships of Platanistidae (Indian River dolphins) and Lipotidae (Yangtze River dolphins) (Fig. 1).

The results of the current partitioned Bayesian analysis using a relaxed molecular clock (Fig. 3) do not fully resolve the relationships of the river dolphin lineages. Platanistidae is strongly placed in a clade exclusive of physeterids (sperm whales), but its relationship with Ziphiidae (beaked whales) and other odontocetes (Fig. 3, Clade I) is unclear as the branch leading to Ziphiidae + Clade I is poorly supported. The situation with Lipotidae is very similar. Although strongly placed in a clade exclusive of Physeteridae, Ziphiidae, and Platanistidae (Clade I), their relationships with the other river dolphins (Iniidae and Pontoporidae) are only weakly supported.

Given that we were unable to unambiguously resolve all of the odontocete relationships with confidence, how does this study further our understanding of cetacean evolution? First, we note that lack of confidence in phylogenetic reconstructions is preferable to strong support for erroneous reconstructions [28]. Yet, more importantly, because we used fossil calibration constraints and a relaxed molecular clock, the branch lengths of our phylogeny are in units of time. Inspection of the branches critical to understanding the phylogenetic placement of both Platanistidae and Lipotidae and their closely related lineages reveals that they diverged within a very narrow time frame (Fig. 3). Indeed, the branch leading to the clade of non-platanistid river dolphins (branch J) is almost indistinguishable from zero. These results are indicative of a rapid radiation, where lineages diversified so quickly that very few DNA character changes had sufficient time to evolve and become fixed. Rapid radiations are notoriously difficult to reconstruct (e.g., [41-44]), especially those that occurred deeper in time (because synapomorphies are more likely to be lost due to stochastic changes).

If the hypothesis that rapid radiation accounts for the phylogenetic uncertainty in odontocete evolutionary relationships is true, what influenced this radiation? Hamilton et al. (2001) hypothesized that the extreme fluctuations of sea levels in the Middle Miocene promoted diversification in all river dolphin lineages by permitting ecological diversification when seas inundated formerly terrestrial habitat [11]. However, this study also assumed that these lineages diversified ~5 million years more recently than those estimated by our study. Given that these extreme sea level changes occurred throughout the Lower and Middle Miocene [45,46], this hypothesis may explain the rapid diversification of Lipotidae and Iniidae + Pontoporidae, which diverged some time between ~25 to 17 Mya (Table 1; Fig. 3). However, our results indicate that the lineage leading to extant (clade G) platanistids, ziphiids, and their relatives diverged in the Middle to Late Oligocene, ~32 to 23 Mya (Table 1; Fig. 3) thus precluding Middle Miocene sea level change explanation for this radiation.

The Oligocene is characterized as an "icehouse" period where global environments were significantly cooler than average [47,48] and was also characterized by extensive expansion of polar ice [49]. Expansion of the polar ice and more extreme ocean thermal gradients both vertically (depth) and horizonally (area) may have resulted in a concomitant expansion of food resources, and have been hypothesized to explain that radiation of cetaceans also seen in the fossil record [50-52]. The relationships of fossil odontocete taxa are the subject of ongoing revision, and it is therefore difficult to determine the phylogenetic relationships of these taxa with any degree of confidence. Nonetheless, there are multiple distinct Oligocene odontocete lineages represented in the fossil record that have subsequently gone extinct (see [32] for review).

Despite partially ambiguous results, our analysis nonetheless provides insight into the evolution of river dolphins. Extant river dolphins are relict lineages whose adaptation to riverine habitats permitted their survival for many millions of years [10]. Additionally, our results reject the hypothesis of a single ecological shift to riverine habitats in the river dolphins [12], and instead support multiple shifts [10,11,13,17,30]. Based on the geographical distribution of Lipotidae (China) and Pontoporidae + Iniidae (South America), and extinct *Parapontoporia *[53] (Pacific North America), a sole invasion of the riverine environment by their most recent common ancestor is unlikely [12]. Based on our phylogenetic inference, we predict three older invasions into the riverine habitats by 1) platanistids in the Early to Late Oligocene, 2) lipotids in the Early Miocene, and 3) the most recent common ancestor of Iniidae and Pontoporidae occurring in the Early to Middle Miocene in addition to a relatively recent reinvasion into the marine coastal habitat by the La Plata river dolphin.

The evolutionary history picture is much clearer within the Delphinoidea. Numerous studies have disagreed on the interrelationships of Delphinidae (dolphins), Monodontidae (narwhals and belugas), and Phocoenidae (porpoises), with some studies supporting the sister relationship of Delphinidae and Phocoenidae [5,12,30], Monodontidae and Phocoenidae [10,14,17,54] or are unable to resolve the relationships [8,11,13,55]. Our results strongly support the Monodontidae + Phocoenidae hypothesis. Moreover, our divergence date estimates (Table 1; Fig. 3) indicate that the major extant lineages radiated in the Middle Miocene, a time, as noted above, when marine environments underwent cyclical diversification and contraction.

The application of recent advances in modeling DNA evolution and use of a large (over 10 kb) data set could not resolve, with significant support, the closest living relatives of Platanistidae or Lipotidae. However, given our results, we propose a potential explanation of why these relationships are so difficult to reconstruct – these lineages are part of a rapid radiation. Thus, future phylogenetic efforts should focus on multiple, independently evolving loci in the nuclear genome. A larger number of independent loci would provide additional phylogenetically informative characters and permit the use of direct statistical tests of the rapid radiation hypothesis (e.g., [43]) as these tests have little power when used with a single locus (e.g., mitochondrial DNA).

### The taxonomy of Delphinidae underestimates its diversity

As with the odontocetes, there has been considerable phylogenetic uncertainty among species within the Delphinidae. Our phylogenetic analyses including seven newly collected complete mitochondrial genomes strongly reject the monophyly of the genera *Stenella *and *Tursiops*. *Stenella coeruleoalba *and *T. aduncus *form a strongly supported clade with *D. capensis *(however, the sister relationship between *St. coeruleoalba *and *D. capensis *is marginally insignificant [PP = 0.94]) and there is strong support for the exclusion of *T. truncatus *and *St. attenuata *from this clade (Fig. 3). These results mirror those of LeDuc et al.'s (1999) study using the cytochrome *b *gene and all three species of *Delphinus *[9]; this study also inferred a trichotomy of *Delphinus *spp., *St. coeruleoalba*, and *T. aduncus*. However, unlike LeDuc et al. (1999) [9] (Fig. 2b) and May-Collado and Agnarsson (2006) [17] (Fig. 2a) and Caballero et al. (2008) [19], our results are able to resolve the placement of *Sousa chinensis *and *St. attenuata *with very strong support (PP = 1.0).

The monophyly of the genera *Tursiops *and *Stenella *has been questioned for more than a century [20,56,57]. There exists a complex of cranial characters not shared by all species of the genus *Stenella*, some of which may actually be more closely related to *Tursiops *or *Delphinus *than to their congeners [56]. To add to the confusing phylogenetic signal from morphological data, some osteological studies suggest a greater affinity between the two bottlenose dolphins (*T. aduncus *and *T. truncatus*) than between *T. aduncus *and any *Stenella *species [23].

Nonetheless, the results of our phylogenetic analysis are clear – *Tursiops *and *Stenella *are not monophyletic and the current taxonomy of Delphinidae masks potentially interesting patterns of morphological, physiological, behavioral, and ecological evolution. That these two genera are not monophyletic suggests that the morphological similarity among species in *Tursiops *and *Stenella *(as currently defined) could be due to adaptive convergence, primitive retention of ancestral body form, reversal to ancestral body form (in *St. coeruleoalba*), or a combination of these factors. Unfortunately, the current taxon sampling of Delphinidae prohibits us from testing these two scenarios (and indeed, making taxonomic revisions), but it certainly deserves intense scrutiny in future phylogenetic and evolutionary analyses of Delphinidae.

Another possible explanation for the phylogenetic pattern is mitochondrial introgression. Although no record of the historical geographical distribution of *Tursiops *or *Stenella *species is available, the two extant bottlenose dolphins (*Tursiops*) are sympatric across parts of their range, particularly around the Penghu Archipelago which is situated midway across the Taiwan Strait [58]. Similarly, both *St. coeruleoalba *and *St. attenuata *are cosmopolitan in tropical and temperate waters around the world. Considering numerous cases of hybridization in captivity or in the wild among inter-species and inter-genera in the family Delphinidae, it is possible that ancient mitochondrial introgression may confound the true evolutionary relationships [9]. Although answering this question with certainty will require collection of multiple nuclear loci, our results demonstrate that recent introgression, at least, is an unlikely explanation for our results. Although closely related, all of our sampled delphinid species are genetically distinct. In other words, there are genetic changes on the terminal branches leading to each species (i.e., autapomorphies). If there was recent mitochondrial introgression, we would expect the introgressed species to be genetically identical (or nearly so) to another species.

## Conclusion

Our phylogenetic analysis of complete mitochondrial genomes using recently developed models of rate autocorrelation resolved the phylogenetic relationships of the major Cetacean lineages with a high degree of confidence. Moreover, our estimation of molecular divergence dates allowed us to construct hypotheses explaining the lack of resolution of the river dolphin lineages. Furthermore, by collecting and analyzing seven new mitochondrial genomes, we provide strong evidence that the generic taxonomy of Delphinidae underestimates the evolutionary history of the group and that the genera *Tursiops *and *Stenella *are not monophyletic. This result has important implications for the morphological evolution (and potentially physiological, behavioral, and ecological evolution) within these genera and indicates adaptive convergence, retention of ancestral body form, or both.

## Methods

### Sample collection and location

We sequenced complete mitochondrial (mt) genomes for seven species representing two species of *Stenella *and *Tursiops *as well as potentially closely related genera (Table 2). Complete mt-genome sequences from these specimens are deposited in GenBank under accession numbers EU557091–EU557097. Muscle tissue was preserved in a solution of 0.25 M disodium EDTA, 20% DMSO, and saturated with NaCl [59], and blood was stored in a vacutainer containing 1.5 mg/ml potassium-EDTA. All tissues were subsequently frozen at -20°C. The voucher specimens were preserved in 95% ethanol at Nanjing Normal University. Fourteen additional mt-genomes for other cetartiodactyla (artiodactyls and cetaceans) were obtained from the GenBank and included in our analyses (Table 2).

**Table 2:** Cetartiodactyla mitochondrial genomes analyzed in this study.

Superfamily	Family	Scientific name	Common name	Sampling location	GenBank accession no.
Delphinoidea	Delphinidae	*Tursiops aduncus*	Indo-Pacific bottlenose dolphin	Dongshan, Fujian Province, China	EU557092, this study
		*Tursiops truncatus*	Common bottlenose dolphin	Polar and Oceanic Park, Shandong Province, China	EU557093, this study
		*Delphinus capensis*	Long-beaked common dolphin	Leqing, Zhejiang Province, China	EU557094, this study
		*Stenella coeruleoalba*	Striped dolphin	Dongshan, Fujian Province, China	EU557097, this study
		*Stenella attenuata*	Pantropical spotted dolphin	Dongshan, Fujian Province, China	EU557096, this study
		*Sousa chinensis*	Indo-Pacific humpbacked dolphin	Xiamen, Fujian Province, China	EU557091, this study
		*Lagenorhynchus albirostris*	White-beaked dolphin	-	AJ554061 (Arnason et al., 2004)
		*Grampus griseus*	Risso's dolphin	Dongshan, Fujian Province, China	EU557095, this study
	Monodontidae	*Monodon monoceros*	Narwhal	-	AJ554062 (Arnason et al., 2004)
	Phocoenidae	*Phocoena phocoena*	Harbor porpoise	-	AJ554063 (Arnason et al., 2004)
Inioidea	Pontoporiidae	*Pontoporia blainvillei*	La Plata river dolphin	-	AJ554060 (Arnason et al., 2004)
	Iniidae	*Inia geoffrensis*	Amazon river dolphin	-	AJ554059 (Arnason et al., 2004)
Lipotoidea	Lipotidae	*Lipotes vexillifer*	Yangtze river dolphin	Jiangyin, Jiangsu Province, China	AY789529 (Yan et al., 2005)
Platanistoidea	Platanistidae	*Platanista gangetica*	South Asian river dolphin	-	AJ554058 (Arnason et al., 2004)
Ziphioidea	Ziphiidae	*Hyperoodon ampullatus*	North Atlantic bottlenose whale	-	AJ554056 (Arnason et al., 2004)
Physeteroidea	Physeteridae	*Physeter catodon*	Sperm whale	Hvalfjordur, Iceland	NC_002503 (Arnason et al., 2000)
Balaenopteroidea	Balaenopteridae	*Megaptera novaeangliae*	Humpback whale	Antarctic ocean	AP006467 (Sasaki et al., 2005)
		*Balaenoptera physalus*	Fin whale	-	X61145 (Arnason et al., 1993)
Hippopotamoidea	Hippopotamidae	*Hippopotamus amphibius*	Hippopotamus	-	NC_000889 (Ursing and Arnason, 1998)
Bovoidea	Bovidae	*Bos taurus*	Cow	-	NC_006853 (Lowe and Eddy, 1997)
		*Ovis aries*	Sheep	-	NC_001941 (Hiendleder et al.,1998)

### Laboratory protocols

Total genomic DNA from muscle tissue was extracted with a standard phenol/chloroform procedure followed by ethanol precipitation [60]. For blood, we used the DNeasy Blood Extaction Kit (Qiagen) in a separate laboratory facility. In order to avoid amplifying nuclear pseudogene copies, approximately 7 kb and 9 kb fragments were amplified using the same long-range PCR protocol as Sasaki et al. [34]. These large mtDNA products were subsequently used as template for short-range PCR of 0.5–1.5 kb using conserved primers (see Additional file 1). PCR was set up with approximately 100 ng of template DNA, 10 × PCR buffer, 2–3 mM MgCl_2_, 1.5 uM each primer, 1 mM dNTPs, 2 U of rTaq (Takara, Japan) and increased to a reaction volume of 50 ul with ultrapure water. Amplification conditions involved an initial denaturing step at 95°C for 5 min, followed by 35 cycles of 95°C for 40 s, 50–55°C for 40 s and 72°C for 60 s, with a final extension step at 72°C for 10 min. Negative controls were run for all amplifications to check for possible contamination. Amplified products were isolated by gel electrophoresis, then excised and purified on Wizard^® ^SV Gel and PCR Clean-Up System (Promega, USA). Sequencing reactions were performed according to the manufacturer's protocols, run on a 3100 or 3700 ABI DNA sequencer, and sequenced in both forward and reverse directions.

### Phylogenetic analyses

Overlapping contigs were compiled using SeqMan II (DNASTAR, Inc. Madison, WI) to generate continuous sequences. To minimize alignment error inherent in phylogenetic analysis of RNA data, we analyzed only protein-coding genes encoded by the mitochondrial heavy-strand, for a total of 10,803 nucleotides from 12 genes (ND6 was excluded to make the data set comparable to existing mt-genome studies).

We employed Bayesian phylogenetic analysis to infer the phylogeny of the sampled cetacean mt genomes. Bayesian analysis incorporates the likelihood equation and models of nucleotide evolution, and has been repeatedly shown to outperform other phylogenetic methods (e.g., [25,26,28]). However, unlike maximum likelihood, model parameters (e.g., tree topology, branch lengths, substitution rates, base frequencies, site rate heterogeneity) in Bayesian analysis are sampled according to their posterior probability [61-64]. Thus, Bayesian analysis inherits many of the attractive qualities of maximum likelihood, but with the added benefit of simultaneously inferring the posterior probabilities of model parameters. In the case of the tree topology, these posterior probabilities can be interpreted as the probability that the clade is correct, given the model [65]. Bayesian analysis is also useful because the use of posterior probabilities allows for very simple hypothesis testing (e.g., the posterior probability of alternative hypotheses can be calculated). Furthermore, the recent development of "relaxed" molecular clocks permits the modeling of autocorrelated rates of evolution [31], thereby relaxing the assumption that every lineage evolves at the same rate and permitting the simultaneous estimated of divergence between lineages.

All phylogenetic analyses were conducted using BEAST v1.4.8 [33] incorporating a lognormal relaxed molecular clock to model rate autocorrelation. Incorporating autocorrelation requires some *a priori *estimate of molecular rates. Because locus-specific rates of evolution are rarely known, one can estimate these rates (i.e., "calibrate" the rate) for one or more lineages using fossil constraints. Modern Bayesian methods allow for the incorporation of a prior distribution of ages ("age constraints"), and thus uncertainty, into these fossil calibrations and ultimately, autocorrelation estimates. Fortunately, Cetacea has an extensive fossil record (see [32]) permitting the use of multiple calibration constraints. All fossil calibrations were lognormal distributions of ages as they best represent the information that fossils provide about the age of a clade (i.e., a lineage most likely diverged around the age of the fossil lineage, but may have diverged earlier [66]). Note that, unlike calibration *points*, these are *distributions *of ages that permit the explicit incorporation of error in the fossil age and times that lineages actually diverged.

We chose prior age distributions so that the youngest age of the distribution corresponded with the youngest possible age of the fossil (i.e., the youngest possible age that lineage existed). We chose a standard deviation of the distribution so that 95% of the distribution fell within the geological time period of the fossil (i.e., 5% of the tail extended into older ages). For example, if a fossil was from Late Miocene deposits (11.2 – 6.5 million years ago [Mya]), we would choose a distribution so that the youngest age of the distribution was 6.5 and 95% of the distribution fell within 11.2 to 6.5 Mya. We used distributions of fossil ages that spanned the entire geological age of the strata in which the fossil was discovered, unless the author provided a more precise fossil age. We used the following fossils as calibration age constraints:

1. The age of the divergence between Hippopotomidae and Cetacea was calibrated using the Ypresian (Eocene: 55.8 – 48.6 Mya) fossil *Pakicetus *[67,68]. We chose a lognormal distribution so that the earliest possible sampled age corresponds to 48.6 Mya and the older 95% credible interval (CI) encompasses the beginning of the Ypresian (55.8 Mya) (standard deviation = 1.2). When estimating divergence dates, calibration ages of divergences close to the root are extremely important (e.g., [66,69]). We therefore enforced the monophyly of this clade in accordance with numerous phylogenetic analyses that have inferred this relationship [4,5,17,70-78].

2. The divergence between Mysticeti (baleen whales) and Odontoceti (toothed whales) was calibrated using the earliest record of mysticetes from the Eocene/Oligocene boundary (see [32] for a review). The Eocene/Oligocene boundary was a critical period in the evolution of both plants and animals (see [79]) and there has been debate about when this transition occurred (38 – 33.5 Mya; [80]). We chose a lognormal distribution so that the earliest possible sampled age corresponds to 33.5 Mya and the older 95% credible interval (CI) encompasses the beginning of the Late Eocene (40 Mya) (standard deviation = 1.138).

3. The age of the root of crown Odontoceti was calibrated using the earliest record of a physeterid (sperm whale) the Late Oligocene *Ferecetotherium *([81]). We chose a lognormal distribution so that the earliest possible sampled age corresponds to 23.7 Mya and the older 95% credible interval (CI) encompasses the beginning of the Late Oligocene (30 Mya) (standard deviation = 1.119).

4. The divergence between Iniidae (the Amazon River dolphin) and Pontoporidae (La Plata River dolphin) was calibrated using the earliest record of a pontoporid, the Middle Miocene *Brachydelphis *[37,82]. We chose a lognormal distribution so that the earliest possible sampled age corresponds to 11.2 Mya and the older 95% CI encompasses the beginning of the Middle Miocene (16.6 Mya) (standard deviation = 1.025).

5. The divergence between Phocoenidae (porpoises) and Monodontidae (narwhals) was calibrated using the earliest record of a phocoenid, the Late Miocene *Salumiphocaena *[83]. This fossil is approximately 10–11 million years old [83], and we chose a lognormal distribution so that the earliest possible sampled age corresponds to 10 Mya and the older 95% CI encompasses the beginning of the Late Miocene (11.2 Mya) (standard deviation = 1.138).

Each BEAST analysis consisted of 2 × 10^7 ^generations with a random starting tree, birth-death default priors (with the exception that we used a uniform [0, 100] prior distribution for the GTR substitution rates), sampled every 1000 generations. Previous studies have demonstrated that applying separate models of nucleotide evolution to specific subsets of nucleotide data (i.e., "partitioned" or "mixed-model" analyses) improves phylogenetic reconstruction [84-86]. We therefore partitioned the data *a priori *by codon position for the combined protein coding gene data set (three total partitions). To determine convergence, we constructed cumulative posterior probability plots for each clade using the *cumulative *function in AWTY [87]. Stationarity was assumed when the cumulative posterior probabilities of all clades stabilized. These plots indicated that excluding the first two million generations as burn-in was sufficient, and the frequency of inferred relationships in the remaining trees represented estimated posterior probabilities. To decrease the chance of reaching apparent stationarity on local optima, two separate analyses were performed. Posterior probability estimates for each clade were then compared between the two analyses using a scatter-plot created by the *compare *command in AWTY. If posterior probability estimates for clades were similar in both analyses, the results of the analyses were combined. Posterior probabilities ≥ 0.95 are considered statistically significant (i.e., "strong") clade support [65].

## Authors' contributions

YX participated in the design of the study and performed most of the laboratory work. MCB and YX conducted all phylogenetic analyses and prepared the manuscript. SX helped to sequence the mitochondrial genome of the Indo-Pacific humpbacked dolphin. KZ helped to improve the manuscript. GY made the overall design of the study, and helped to draft and improve the manuscript. All authors read and approved the last version of the manuscript.

## Supplementary Material

Additional file 1 **Primers used for amplifying and sequence the Delphinidae mtDNA in this study.** This table provides names and relevant sequence information of the primers used for amplifying and sequencing the complete mtDNA genome of seven delphinids. Click here for file
